# Synthesis of Titanium Dioxide-Based Pigments with Co-Precipitated Phosphate

**DOI:** 10.3390/ma19143052

**Published:** 2026-07-15

**Authors:** Hiroaki Onoda, Yurina Kita

**Affiliations:** Department of Biomolecular Chemistry, Faculty of Science and Technology, Kyoto Prefectural University, 1-5, Shimogamo Nakaragi-cyo, Sakyo-ku, Kyoto 606-8522, Japan

**Keywords:** white pigment, titanium dioxide, photocatalytic activity

## Abstract

Titanium dioxide is used as a white pigment in cosmetics and other products, but it possesses photocatalytic activity and has become problematic for decomposing sebum when exposed to sunlight. Consequently, titanium phosphate has been investigated as a new white pigment, with expectations for its biocompatibility. However, this pigment had the drawback of having particles that were too large. Therefore, in this study, as a new process for cosmetic pigments, we prepared samples where titanium hydroxide and titanium phosphate were coprecipitated, and we attempted to create a mixed material of titanium oxide and titanium phosphate by heating. This method aimed to suppress photocatalytic activity and produce a novel pigment with particle sizes suitable for cosmetics. With this method, thorough rinsing with water is necessary to remove the sodium sulfate. Even in samples with a low phosphorus ratio of Ti/P = 10/1, photocatalytic activity was sufficiently suppressed. Under this Ti/P = 10/1 condition, the drawback of excessively large particle sizes typically found in phosphate pigments is not particularly noticeable. This study is expected to contribute to the development of new white pigments for use in cosmetics.

## 1. Introduction

Titanium dioxide is used as a white pigment in cosmetics alongside zinc oxide [1,2,3,4]. Their advantages include having undergone safety testing for skin contact and being available in various particle sizes. On the other hand, titanium dioxide is well known as a material with photocatalytic activity [5,6,7,8]. When cosmetics containing titanium dioxide are applied to the skin, exposure to ultraviolet light causes sebum to break down. Methods to suppress this photocatalytic activity for use in cosmetics and other products have been investigated, but it is difficult to suppress photocatalytic activity using a simple method [9,10].

Phosphates are highly biocompatible materials, as evidenced by the fact that the primary components of bones and teeth are a type of calcium phosphate [11,12,13,14]. In addition, the biocompatibility of titanium phosphate glass has been investigated [15,16]. Cosmetics are materials that come into prolonged contact with the skin, so biocompatibility that does not cause allergies or other adverse reactions is desirable. Titanium phosphate has been investigated as a new white pigment to replace titanium dioxide [17]. While this compound did not exhibit photocatalytic activity, its drawback was that the particle size was too large. Using pigments with large particle sizes is thought to make cosmetics prone to cracking [18]. Furthermore, if the particles are too small, the pigment can remain in pores and continuously burden the skin, making it unsuitable [19]. Therefore, cosmetic white pigments are required to lack photocatalytic activity and to be sub-micrometer-sized particles. Pigments formed by reacting titanium dioxide surfaces with phosphates have been reported [20]. These pigments possess appropriate particle sizes, but their photocatalytic activity has been suppressed while still being retained. In addition, white pigments co-precipitated with cerium phosphate have been reported to suppress the oxidation catalytic activity of cerium oxide [21]. In this research, a cerium hydroxide–cerium phosphate mixture was precipitated in aqueous solution, then filtered and heated to obtain cerium oxide–cerium phosphate pigment. This method demonstrated the potential to prepare samples more easily and to suppress catalytic activity.

In this study, titanium dioxide pigments with coprecipitated phosphates were prepared by obtaining hydroxide-phosphate mixed samples via the aqueous precipitation method and then heating them. The objective of this research is to obtain pigments with suppressed photocatalytic activity and particles of appropriate size.

## 2. Materials and Methods

The 0.5 mol/L of titanium sulfate solution and 0.5 mol/L of phosphoric acid were mixed in Ti/P molar ratios of 10/1, 10/3, 10/5, 10/7, and 10/9. This solution was adjusted to pH 6 using about 8 mol/L of sodium hydroxide solution. All chemicals were commercially pure (FUJIFILM Wako Pure Chemicals Corporation, Osaka, Japan) and were used without further purification. The formed precipitate was filtered and washed twice with sufficient water. A portion of the dried sample was heated at 300, 500, and 700 °C for one hour under air condition.

The crystal structures and chemical bonds of these materials were analyzed using X-ray diffraction (XRD) patterns and infrared (IR) spectra, respectively. XRD patterns were recorded on an X-ray diffractometer (MiniFlex, Rigaku Corporation, Akishima, Japan) using monochromatic CuKα radiation (30 kV, 15 mA, 3°/min, step size: 0.02º). IR spectra of the samples were recorded by KBr disk method (Resolution: 4 cm^−1^, 16 times scanned) using a HORIBA FT-IR 720 (HORIBA Corporation, Kyoto, Japan).

The color of the pigments was evaluated using a TES135 plus color analyzer (TES Electrical Electronic Corp, Taipei, Taiwan). The L* value indicates the whiteness of the sample powder, with 100 representing white and 0 representing black [22,23]. The a* value indicates the redness of the material, with positive values corresponding to red and negative values corresponding to green. The b* value indicates the yellowness of the sample, with positive values corresponding to yellow and negative values corresponding to blue.

Particle size distributions were measured using a centrifugal precipitation-type analyzer (SA-CP3L; Shimadzu Corp., Kyoto, Japan). The density used for measurement was set at 3.7 g/cm^3^, based on titanium phosphate at 3.58 g/cm^3^, titanium hydroxide at 3.5–4.0 g/cm^3^, and titanium oxide at 3.9 g/cm^3^.

Photocatalytic activity was evaluated by the decomposition of methylene blue (MB) ethanol solution under UV irradiation at 365 nm. A 0.01 g sample of each material was suspended in 4 mL of a 1.0 × 10^−5^ mol/L MB solution. Using a UV-Vis spectrophotometer, the absorbance at 655 nm was measured at 20-min intervals for up to 120 min.

Samples heated at 700 °C (0.1 g) were left to stand for 24 h in 0.1 wt% H_2_SO_4_ or NaOH aqueous solution (100 mL), then filtered and dried. The reason samples heated to 700 °C were used here was that it was determined with certainty that they had undergone a transformation from titanium hydroxide to titanium oxide. The acid and base resistance of the samples was evaluated based on recovery percentage and color difference ΔE before and after treatment.

ΔE* = [(L* _after_ − L* _before_)^2^ + (a* _after_ − a* _before_)^2^ + (b* _after_ − b* _before_)^2^]^1/2^ (1)

## 3. Results and Discussion

Prior to this experiment, Ti/P = 10/1 samples were prepared at pH 3, 5, 6, 7, and 9. Based on the results, samples were prepared at pH 6 in this study. Furthermore, since initial studies showed high proportions of sodium sulfate in the precipitate samples, this study employed washing with a larger volume of water. Furthermore, the samples were heated at 300, 500, and 700 °C, and the effects of heating were examined. Samples heated at 300 °C and 500 °C exhibited weak XRD peaks for titanium dioxide. The unheated sample contained a high proportion of particles measuring 10–20 µm in size, and heating resulted in smaller particle sizes. This was attributed to the difference in particle size between titanium hydroxide and titanium dioxide. Based on the above, this study primarily focused on samples heated at 700 °C.

**Figure 1:**
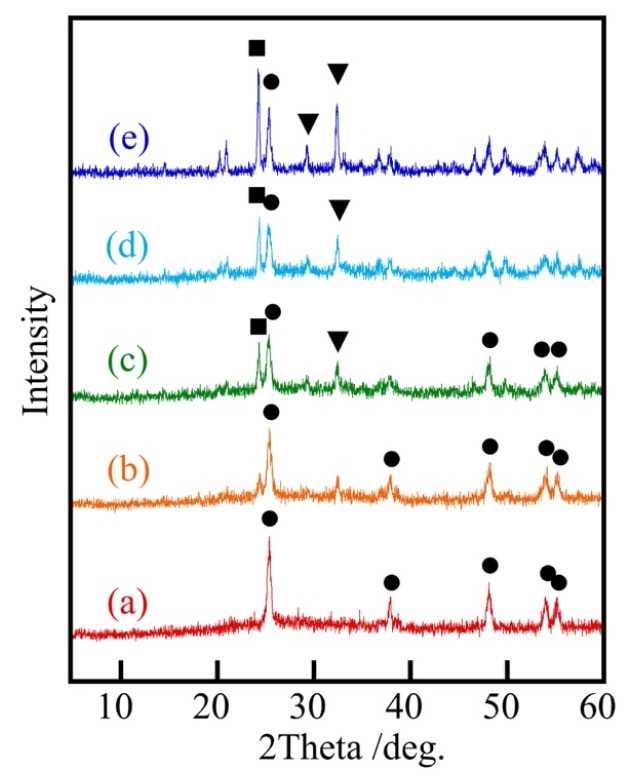
XRD patterns of samples prepared at various Ti/P ratios (700 °C), (**a**) 10/1, (**b**) 10/3, (**c**) 10/5, (**d**) 10/7, (**e**) 10/9, ●; TiO_2_, ◼️; TiP_2_O_7_, ▼; Na_2_SO_4_.

The samples prepared in this study were amorphous when unheated. This was thought to be because the samples were obtained by precipitating the compounds in aqueous solution, preventing the molecules from arranging in a regular manner. Figure 1 shows the XRD patterns of samples prepared with various Ti/P ratios and then heated at 700 °C. All samples exhibited titanium dioxide peaks [24]. The peaks at 25.4, 37.9, 48.1, 54.0, and 55.1° are due to the (101), (004), (200), (105), and (211) crystal planes. In samples with a higher phosphorus content, TiP_2_O_7_ peaks were observed [25]. This compound has a Ti/P ratio of 1/2, exhibiting a significantly higher phosphorus content than the synthesis conditions used in this study. This compound is considered relatively easy to form, and its peak intensity increases as the phosphorus content rises. Additionally, samples under conditions with a high phosphorus content showed a detectable peak for sodium sulfate [26]. It was confirmed that sodium sulfate, which could not be completely removed even by washing with a large amount of water, was present in the sample. It was thought that the precipitation of phosphate ions made sulfate ions more likely to precipitate, resulting in a higher concentration of sulfate ions in the sample.

**Figure 2:**
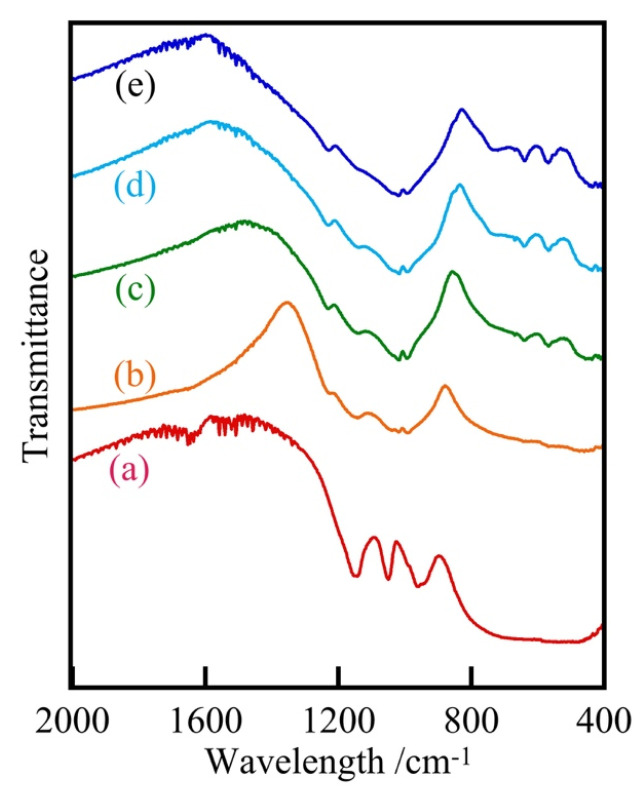
IR spectra of samples prepared at various Ti/P ratios (700 °C), (**a**) 10/1, (**b**) 10/3, (**c**) 10/5, (**d**) 10/7, (**e**) 10/9.

Figure 2 shows the IR spectra of samples prepared with various Ti/P ratios and then heated at 700 °C. The broad peak at 400–800 cm^−1^ originates from oxides, while the peak near 1000 cm^−1^ is due to P-O stretching of phosphate ions [27,28]. As the phosphorus content increased, the oxide peak weakened while the phosphate peak strengthened. This peak shift is judged to correspond to the ratio of oxides and phosphates present in the sample.

Table 1 shows the L*a*b* values of samples prepared with various Ti/P ratios and then heated at 700 °C. All samples exhibited L* values of 98 or higher, corresponding to high whiteness. The a* and b* values were all close to zero, indicating that the sample did not possess any specific color.

**Figure 3:**
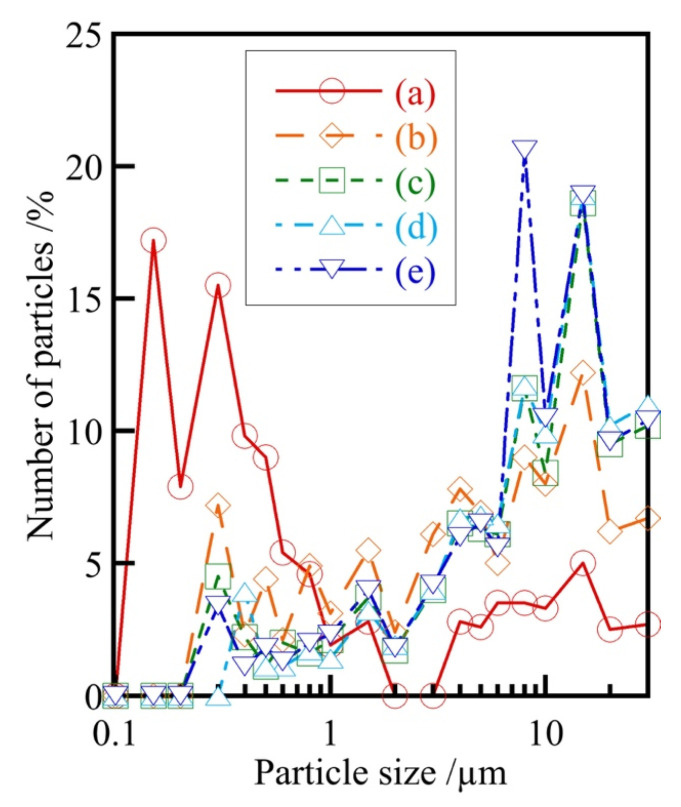
Particle size distribution of samples prepared at various Ti/P ratios (700 °C), (**a**) 10/1, (**b**) 10/3, (**c**) 10/5, (**d**) 10/7, (**e**) 10/9.

Figure 3 shows the particle size distribution of samples prepared at various Ti/P ratios and then heated at 700 °C. The sample prepared at Ti/P = 10/1 exhibited a significantly higher proportion of smaller particles compared to the other samples. This corresponds to previous reports indicating that titanium phosphate possesses relatively large particle sizes. Specifically, it was considered that the Ti/P = 10/1 sample had a low phosphate content, which prevented the particle size from increasing.

**Figure 4:**
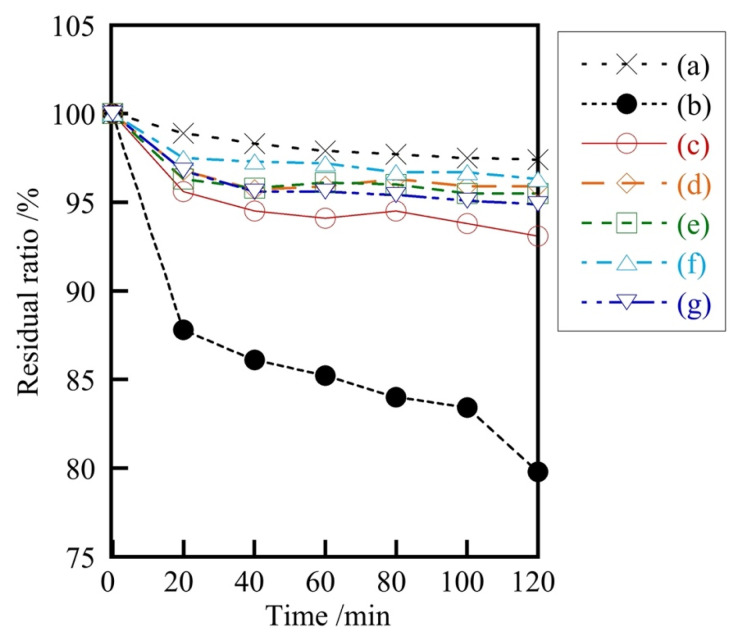
Photocatalytic activity of samples prepared at various Ti/P ratios (700 °C), (**a**) blank, (**b**) TiO_2_, (**c**) 10/1, (**d**) 10/3, (**e**) 10/5, (**f**) 10/7, (**g**) 10/9.

Figure 4 shows the photocatalytic activity of samples prepared with various Ti/P ratios and then heated at 700 °C. For use as a white pigment in cosmetics, it is desirable that the photocatalytic activity of the sample be low. Compared to titanium dioxide, the samples prepared in this study exhibited suppressed photocatalytic activity. This suppression was sufficiently pronounced even in samples with relatively low phosphorus content.

Table 2 shows the acid and base resistance of samples prepared at various Ti/P ratios and then heated at 700 °C. The recovery rate of all samples after immersion in acid or base solution was 50–70%, indicating moderate acid and base resistance. ΔE is approximately 3 or less, indicating that the color does not change even when immersed in acid or base solution. When used outdoors as pigments in paints and similar products, a recovery rate of 80% or higher and a ΔE value of 3 or less are required; however, the samples produced in this study fell slightly short of these standards. However, since cosmetic white pigments are not intended for long-term use in strongly acidic or strongly basic conditions, the values obtained for the samples in this study were sufficient.

## 4. Conclusions

Titanium dioxide coprecipitated with phosphate was proposed as a new white pigment by heating a mixed hydroxide–phosphate precipitate using an aqueous solution. This method requires sufficient water washing to remove sodium sulfate. Compared to conventional white pigments, the distinctive characteristics of this pigment are as follows. (1) Even in samples with a low phosphorus ratio of Ti/P = 10/1, photocatalytic activity is sufficiently suppressed. (2) Under Ti/P = 10/1 conditions, the weakness of excessively large particle sizes observed in phosphate pigments does not become significantly apparent. This pigment has the potential to inhibit the breakdown of sebum caused by exposure to sunlight and provide a smooth application; ultimately, it contributes to the development of new cosmetics.

## Figures and Tables

**Table 1:** L*a*b* values of samples prepared at various Ti/P ratios (700 °C).

Ti/P	L*	a*	b*
10/1	98.7	0.76	1.81
10/3	98.8	−0.01	3.20
10/5	99.6	−1.08	1.55
10/7	99.3	−0.10	1.16
10/9	99.2	−0.83	2.45

**Table 2:** Acid and base resistances of samples prepared at Ti/P ratios (700 °C).

	Acid		Base	
Ti/P	Yield/%	ΔE	Yield/%	ΔE
10/1	55.8	1.53	53.5	3.02
10/3	67.0	1.93	65.3	2.55
10/5	53.2	1.81	65.5	2.22
10/7	62.7	0.74	62.4	0.63
10/9	69.5	0.95	58.7	0.68

## Data Availability

The raw data supporting the conclusions of this article will be made available by the authors on request.
